# O.5.2-6 Using the Physical Activity Messaging Framework to investigate physical activity messaging preferences in new mothers

**DOI:** 10.1093/eurpub/ckad133.247

**Published:** 2023-09-11

**Authors:** Chloë Williamson, Graham Baker, Paul Kelly

**Affiliations:** Physical Activity for Health Research Centre (PAHRC), Institute for Sport, Physical Education and Health Sciences, University of Edinburgh, Edinburgh, UK; chloe.williamson@ed.ac.uk; Physical Activity for Health Research Centre (PAHRC), Institute for Sport, Physical Education and Health Sciences, University of Edinburgh, Edinburgh, UK; chloe.williamson@ed.ac.uk; Physical Activity for Health Research Centre (PAHRC), Institute for Sport, Physical Education and Health Sciences, University of Edinburgh, Edinburgh, UK; chloe.williamson@ed.ac.uk

## Abstract

**Purpose:**

Evidence suggests PA levels decline during pregnancy and remain low during the postpartum period. PA can reduce risk of excessive gestational weight gain, gestational diabetes, and symptoms of postpartum depression. One way to encourage postpartum to engage in PA is through PA messaging. This study aimed to apply the novel Physical Activity Messaging Framework (PAMF) to develop targeted messaging recommendations for postpartum women.

**Methods:**

Proof-of-concept study. Semi-structured individual interviews were conducted with seven new mothers from North Tyneside and Northumberland (mean age 33.6 years). The interview schedule aligned with sections of the PAMF to explore preferences around: (1) message aim and pathway, (2) message content, and (3) message format and delivery. Interviews were audio recorded and transcribed verbatim. Framework analysis was used to identify trends, similarities, and differences in the data.

**Results:**

Key findings related to the three sections of the PAMF. (1) This study identified important potential aims and working pathways for messages to this group, such as reducing barriers to PA and educating new mums on benefits. (2) Findings included that tone and language used in messages should be empathetic and reassuring, and that messages should be gain-framed. New mums value the social and acute mental health benefits of PA, such as connecting with other mums and relaxing. This study also demonstrated the importance of using realistic and inclusive images in PA messages. New mums may find the 150-minute PA guideline unachievable, with a preference for shorter bouts of PA and clear, practical advice. (3) Social media was identified as an viable delivery platform for new mums, and healthcare providers were identified as important messengers.

**Conclusion:**

This study found the PAMF to be a helpful tool to inform data collection, data analysis and organisation of findings in a study aiming to develop PA messages. Findings relating to message aim and pathway, message content, and message format and delivery were identified, and specific targeted PA messaging recommendations for new mums were provided.

**Funding source:**

This research was conducted as part of CW’s PhD, funded by the University of Edinburgh’s Principal’s Career Development Scholarship, Oct 2018 - Jan 2021.

